# Germ Cell Drivers: Transmission of Preconception Stress Across Generations

**DOI:** 10.3389/fnhum.2021.642762

**Published:** 2021-07-12

**Authors:** Korrina A. Duffy, Tracy L. Bale, C. Neill Epperson

**Affiliations:** ^1^Colorado Center for Women’s Behavioral Health and Wellness, Department of Psychiatry, University of Colorado School of Medicine, Aurora, CO, United States; ^2^Center for Epigenetic Research in Child Health and Brain Development, Department of Pharmacology and Psychiatry, University of Maryland School of Medicine, Baltimore, MD, United States; ^3^Department of Family Medicine, University of Colorado School of Medicine, Aurora, CO, United States; ^4^Center for Women’s Health Research, University of Colorado School of Medicine, Aurora, CO, United States; ^5^Helen and Arthur E. Johnson Depression Center, University of Colorado School of Medicine, Aurora, CO, United States

**Keywords:** stress, trauma, epigenetics, small non-coding RNA, germline, sperm, extracellular vesicles, oocytes

## Abstract

Exposure to stress can accelerate maturation and hasten reproduction. Although potentially adaptive, the trade-off is higher risk for morbidity and mortality. In humans, the intergenerational effects of stress have been demonstrated, but the precise mechanisms are unknown. Strikingly, even if parental stress occurs prior to conception, as adults, their offspring show worse mental and physical health. Emerging evidence primarily from preclinical models suggests that epigenetic programming may encode preconception stress exposures in germ cells, potentially impacting the phenotype of the offspring. In this narrative review, we evaluate the strength of the evidence for this mechanism across animals and humans in both males and females. The strongest evidence comes from studies of male mice, in which paternal preconception stress is associated with a host of phenotypic changes in the offspring and stress-induced changes in the small non-coding RNA content in sperm have been implicated. Two recent studies in men provide evidence that some small non-coding RNAs in sperm are responsive to past and current stress, including some of the same ones identified in mice. Although preliminary evidence suggests that findings from mice may map onto men, the next steps will be (1) considering whether stress type, severity, duration, and developmental timing affect germ cell epigenetic markers, (2) determining whether germ cell epigenetic markers contribute to disease risk in the offspring of stress-exposed parents, and (3) overcoming methodological challenges in order to extend this research to females.

## Introduction

Exposure to chronic stress or trauma, particularly in early life, prompts neural, developmental, physiological, and behavioral changes (Maccari et al., 2014; Cameron and Schoenfeld, 2018; Ellis and Del Giudice, 2019). These changes may be adaptive if they increase the likelihood of surviving long enough to reproduce (e.g., by increasing vigilance to threat) and/or accelerate development in order to reproduce sooner (e.g., by reaching puberty earlier). Evidence in humans shows that early life stress does indeed accelerate development, particularly when the adversity is threat-related (e.g., violence) rather than deprivation-related (e.g., food insecurity) (Belsky, 2019; Sumner et al., 2019). In humans, early life adversity is associated with earlier age of reproductive events – puberty, menarche, parturition, and menopause – but also accelerated aging at the cellular level (Wilson and Daly, 1997; Lawlor et al., 2003; Jorm et al., 2004; Bogaert, 2005; Chisholm et al., 2005; Hardy and Kuh, 2005; Mishra et al., 2007; Pesonen et al., 2008; Nettle, 2010; Belsky et al., 2017; Lei et al., 2018; Magnus et al., 2018; Ridout et al., 2018). Early life adversity and its developmental consequences (i.e., accelerated development and aging) are associated with a wide range of poor mental and physical health outcomes and earlier mortality (Felitti et al., 1998; Golub et al., 2008; Thomas et al., 2008; Jones et al., 2009; Wegman and Stetler, 2009; Kessler et al., 2010; McLaughlin et al., 2010, 2016; Shuster et al., 2010; Ma et al., 2011; Rich-Edwards et al., 2012; Widom et al., 2012; Charalampopoulos et al., 2014; Haycock et al., 2014; Janghorbani et al., 2014; Bellis et al., 2015; Duncan et al., 2015; Rode et al., 2015; Campbell et al., 2016; Chen E. et al., 2016; Lei et al., 2018; Wolkowitz, 2018; Carter et al., 2019). Accelerated development may enhance reproductive fitness in a stressful environment but may result in critical systems developing too quickly (e.g., synaptic pruning of the brain), leading to less-than-optimal development. These costly tradeoffs are best understood in an evolutionary context. Life history theory explains why harsh, unstable, and unpredictable conditions tend to produce a faster life strategy – a “live fast, die young” approach, characterized by accelerated maturation, hastened reproduction, and a shortened lifespan.

Programming of a fast life strategy may begin preconception. In humans, parental preconception trauma is associated with alterations in the hypothalamic-pituitary-adrenal (HPA) stress axis in their children (Yehuda et al., 2007a,b; Bader et al., 2014). This may lead to blunted cortisol levels and enhanced negative feedback, which could prepare offspring for life under stressful conditions. However, these stress-induced adaptations may come at a cost for offspring too. Across a wide range of parental stress exposures – genocide, war, combat, famine, and childhood maltreatment – preconception stress is associated with increased risk for negative mental and physical health outcomes in offspring despite not being directly exposed to the stressor (Yehuda et al., 1998; Dubowitz et al., 2001; Kaati et al., 2002; Pembrey et al., 2006; Dekel and Goldblatt, 2008; Miranda et al., 2011; Field et al., 2013; Veenendaal et al., 2013; Bezo and Maggi, 2015). The observation that parental preconception stress negatively affects offspring may be explained by various social and environmental factors, such as prenatal programming (e.g., exposures *in utero*), disadvantaged environments, poorer parenting behavior, family dysfunction, insecure attachment styles in the parent(s), and parental mental health problems (Dubowitz et al., 2001; Dekel and Goldblatt, 2008; Sharkey, 2008; Miranda et al., 2011; Field et al., 2013; Moog et al., 2016; Cooke et al., 2019). However, emerging evidence in animal studies supports a biological pathway by which parents may pass their preconception stress experiences to their offspring through epigenetic germ cell transmission (e.g., Rodgers et al., 2013, 2015; Dias and Ressler, 2014; Gapp et al., 2014; Chan et al., 2020). The strongest evidence for this comes from studies in male mice. These studies have shown that paternal stress experiences are linked with epigenetic changes associated with sperm and subsequent phenotypic alterations in the offspring (e.g., Rodgers et al., 2013, 2015; Gapp et al., 2014; Chan et al., 2020).

Epigenetic changes modify the expression of a gene without altering its underlying genetic sequence (Jaenisch and Bird, 2003). Because environmental exposures induce epigenetic changes, they allow the organism to flexibly and rapidly adapt to the environment, particularly harsh environments. Several types of key epigenetic changes can be altered by the environment, including DNA methylation, histone modifications, and non-coding RNA (ncRNA).

The evidence that stress can modify germ cell epigenetic markers comes from studies investigating DNA methylation and sncRNA. Although paternal genome DNA methylation marks undergo near complete erasure and reprogramming following fertilization, the epigenome is not entirely reset, suggesting that certain epigenetic patterns could be transmitted to offspring through the effects of sperm DNA methylation (Hackett et al., 2013; Ly et al., 2015). However, exactly which DNA methylation patterns escape erasure or are reprogrammed remains to be elucidated. Nevertheless, it is also plausible that DNA methylation changes in the paternal germline could also be translated into a different type of “signal” at conception that is then perpetuated in embryo development, ultimately resulting in a phenotypically meaningful biological change. However, the mechanisms by which specific methylation changes could be propagated are not known. Until the mechanisms are understood, caution should remain as to whether changes to specific DNA methylation marks detected in sperm can be transmitted to offspring.

Unlike DNA methylation, sncRNA are not required to be a self-perpetuated signal following fertilization or during development as they have an immediate function at conception and, therefore, have emerged as causal agents of stress signal transmission (Meikar et al., 2011; Morgan et al., 2019, 2020; Chan et al., 2020). In brief, sncRNA are short RNA that are less than 200 nucleotides in length and are considered “non-coding” because they typically are not translated into proteins (Marcho et al., 2020). Nevertheless, sncRNA regulate the expression of genes and serve as epigenetic mediators of environmental exposures (Fullston et al., 2013; Huang et al., 2013; Rodgers et al., 2013; Chen Q. et al., 2016; Cropley et al., 2016; de Castro Barbosa et al., 2016; Sharma et al., 2016; Conine et al., 2018). Two types of sncRNA that we will discuss further are microRNA (miRNA) and transfer RNA (tRNA), which are typically found in sperm as tRNA fragments (Kiani and Rassoulzadegan, 2013; Chen Q. et al., 2016; Cropley et al., 2016; Sharma et al., 2016, 2018; Selvaraju et al., 2018).

**FIGURE 1:**
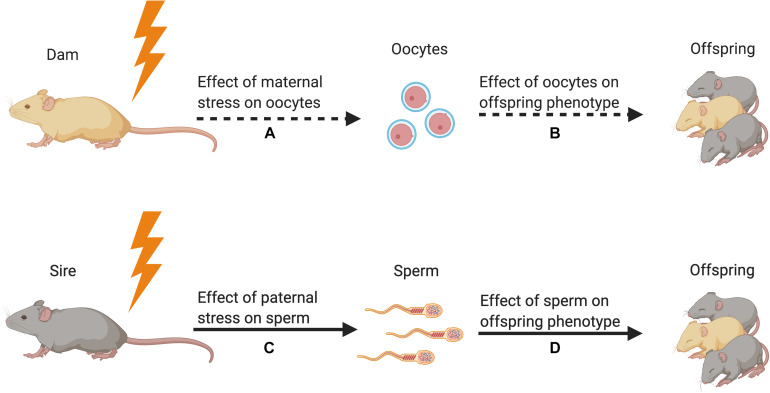
Strength of the evidence for germline transmission of parental stress on offspring phenotype in animal models. **(A)** Only one study has demonstrated an effect of maternal preconception stress on oocytes (Zaidan et al., 2013). This study showed that stress increased corticotropin releasing factor type 1 messenger RNA but did not reveal a change in epigenetic signals. **(B)** No studies have tested for the effect of stress-induced oocyte epigenetic changes on offspring phenotype. **(C)** Many studies have demonstrated that paternal preconception stress affects sperm DNA methylation and small non-coding RNA content (Franklin et al., 2010; Rodgers et al., 2013; Gapp et al., 2014, 2016; Bohacek et al., 2015; Wu et al., 2016; Chan et al., 2020). **(D)** Multiple studies have used *in vitro* fertilization, intracytoplasmic sperm injection, and microRNA microinjection to demonstrate that stress-induced alterations in sperm sncRNA content affect offspring phenotype (Dias and Ressler, 2014; Gapp et al., 2014, 2018; Rodgers et al., 2015; Wu et al., 2016; Chan et al., 2020). Figure created using BioRender.com.

Here, we present evidence that stress is associated with changes in germ cell DNA methylation marks and sncRNA content. These epigenetic changes in the germ cell correspond with alterations in offspring phenotype, with the implication that these epigenetic modifications transmit a “stress” signal to impact offspring development, ultimately altering behavioral, physiological, neural, and/or cognitive outcomes (see Figure 1). The scope of our narrative review is limited to studies examining epigenetic and molecular modifications in germ cells, presented separately for oocytes and sperm (see Table 1 for an overview of the evidence). Currently, the only evidence of epigenetic inheritance is intergenerational, meaning epigenetic transmission is from parent (F0 generation) to offspring (F1), rather than transgenerational, in which epigenetic effects would have to be shown to be propagated to at least the F2 generation. Because the vast majority of studies on preconception stress effects on germ cells have been conducted in males, we focus on sperm studies to highlight factors critical to producing epigenetic changes in sperm and present evidence that these epigenetic changes impact offspring outcomes. Furthermore, because stress-induced changes in sncRNA content in sperm are the epigenetic mechanism most likely to be able to impact offspring (based on our current understanding of which epigenetic markers get transmitted through the germ cell), we narrow in on recent research showing how sperm may acquire these epigenetic changes. Finally, we discuss current knowledge gaps and provocative future research directions.

**TABLE 1:** Summary of studies testing for stress-induced or stress hormone-induced molecular changes in germ cells.

	**Species**	**Stress paradigm**	**Developmental stage**	**Molecular changes**
**Oocytes**				
Zaidan et al., 2013	Mice	Chronic unpredictable stress (1 week)	Adulthood	18.5-fold greater corticotropin-releasing factor type 1 messenger RNA expression (a key component of the stress response)
**Sperm**				
Morgan et al., 2020	Humans	Longitudinally measured perceived stress (6 months)	Adulthood	Five miRNA (let-7f-5p, miR-181a-5p, miR-4454, miR-6765-3p, and miR-12136) and four tRNA (tRNA-Gly-GCC-3-1, tRNA-Lys-CTT-1-1, tRNA-Lys-CTT-2-1, and tRNA-Lys-CTT-4-1) fluctuate in response to perceived stress
Dickson et al., 2018	Humans	Retrospectively measured adverse childhood experiences (from ages 0–18)	Childhood and adolescence	Lower levels of multiple miRNA in the miR-449 and miR-34 families
	Mice	Chronic social instability stress (7 weeks)	Puberty into adulthood (PN28-77)	Five-fold lower levels of miR-449a and miR-34c
Chan et al., 2020	Humans	Longitudinally measured perceived stress (6 months)	Adulthood	Broad changes in miRNA in males recovering from stress compared to those with minimal variation in stress over the 6-month period (determined using principal component analysis)
	Mice	Chronic variable stress (4 weeks)	Puberty (PN28-56)	Dramatic differences in miR-9-3p and miR-34c-5p 12 weeks after stress cessation but not after 1 week
Gapp et al., 2014	Mice	Unpredictable maternal separation combined with unpredictable maternal stress (2 weeks)	Juvenile (PN1-14)	2-4-fold higher levels of miR-200b-3p, miR-672-5p, miR-466c-5p, miR-375-3p, and miR-375-5p (the miR-375 family has been implicated in stress response and metabolic regulation)
Franklin et al., 2010	Mice	Unpredictable maternal separation combined with unpredictable maternal stress (2 weeks)	Juvenile (PN1-14)	Higher DNA methylation for the methyl CpG-binding protein 2 gene (a transcriptional regulator that binds methylated DNA) and cannabinoid receptor 1 gene (associated with emotional behavior in rodents) but lower for corticotropin-releasing factor receptor 2 gene (involved in the stress response)
Bohacek et al., 2015	Mice	Unpredictable maternal separation combined with unpredictable maternal stress (2 weeks)	Juvenile (PN1-14)	Lower DNA methylation at the protein kinase C promotor gene implicated in synaptic plasticity and memory performance
Rodgers et al., 2013	Mice	Chronic variable stress (6 weeks)	Puberty into adulthood (PN28-70) or adulthood (PN56-98)	2-5-fold increases in nine miRNAs (miR-29c, miR-30a, miR-30c, miR-32, miR-193-5p, miR-204, miR-375, miR-532-3p, and miR-698)
Wu et al., 2016	Mice	Daily restraint (2 weeks)	Adulthood (PN58-72)	Two-fold higher DNA methylation of the *Sfmbt* promotor gene involved in gluconeogenesis
		Daily restraint (2 weeks) with daily treatment with glucocorticoid antagonist	Adulthood (PN58-72)	Glucocorticoid antagonist normalized DNA methylation of the *Sfmbt* promotor gene involved in gluconeogenesis
Petropoulos et al., 2014	Mice	Synthetic glucocorticoid administration (5 days)	Adulthood (PN70-75)	20% increase in global non-CpG methylation in sperm 60 days but not 35 days after treatment with synthetic glucocorticoids
Short et al., 2016	Mice	Corticosterone treatment (4 weeks)	Adulthood (PN70-98)	2-4-fold higher levels of three miRNAs (miR-98, miR-144, and miR-190b), which are thought to interact with multiple growth factors
Dias and Ressler, 2014	Mice	Odor fear conditioning to acetophenone (3 days)	Adulthood (2-months old*)	Lower CpG methylation of the *Olfr151* gene that codes for odor receptors activated by acetophenone

*Age in postnatal days not specified. For mice, age at which the stress paradigm occurred is given in both days postnatal (PN) as well as the corresponding developmental stage.

## Transmission of Maternal Preconception Stress to Offspring via the Germ Cell

To our knowledge, only one study to date has examined the effect of preconception stress on oocytes (Zaidan et al., 2013). In this study, F0 adolescent female rats were exposed to chronic unpredictable stress for 7 days, and then stress-exposed and control females either had oocytes extracted or were bred (Zaidan et al., 2013). Reverse-transcription PCR was used to measure corticotropin-releasing factor 1 (CRF1) messenger RNA (mRNA) expression in oocytes and in various brain regions in the offspring. Oocyte expression of CRF1 mRNA was 18.5 times greater in the oocytes of stress-exposed females compared to controls. In the brains of adult offspring from stress-exposed and control dams, group differences in CRF1 expression in the amygdala and frontal cortex emerged but were dependent on the extent to which offspring were exposed to stress in adulthood.

While this study is compelling, it does not provide mechanistic evidence that maternal preconception stress programs offspring outcomes through epigenetic changes to the oocyte. The intrauterine environment and maternal behavior are important confounds that make studying maternal transmission of stress experienced before conception a substantial challenge. These confounds can be reduced but require the embryo to be transferred to the uterus of a non-stressed surrogate female and the neonates to be cross fostered by a non-stressed surrogate mother. The study discussed above did not control for either of these factors.

In humans, testing whether oocytes show epigenetic changes in response to stress is nearly impossible. In the US and in other countries, access to oocytes is extremely limited owing to the fact that federal funds cannot be used to provide compensation for the donation of oocytes for research (Spar, 2007; Safier et al., 2018). As a result, no studies have assessed stress-induced epigenetic changes to human oocytes. Given challenges of studying this in humans, mechanistic research will rely on animal studies for the foreseeable future.

## Transmission of Paternal Preconception Stress to Offspring via the Germ Cell

Studies on paternal preconception stress provide more evidence of epigenetic germ cell transmission than studies on maternal preconception stress. In this section, we first examine evidence across animal models and human studies showing that stress is associated with epigenetic changes in sperm and phenotypic alterations in offspring. Then, we consider how stress hormones and a stress recovery period are necessary to produce epigenetic changes in sperm; we review the mechanistic evidence that sncRNA changes in sperm drive alterations in offspring phenotype, drawing on studies that use methodologies to isolate the effect of sncRNA on offspring outcomes; and, finally, we discuss how extracellular vesicles in the male reproductive tract may affect the sncRNA content of sperm.

### Human Studies

In human studies of male preconception stress on sperm epigenetic changes, research suggests that trauma in childhood and perceived stress in adulthood are associated with changes in the sncRNA content of sperm (Dickson et al., 2018; Morgan et al., 2020). In a cross-sectional study, a higher number of adverse childhood experiences (ACEs; e.g., abuse, neglect, family dysfunction in childhood) correlated with lower levels of multiple miRNA in sperm (Dickson et al., 2018). In the first longitudinal study to measure changes in sperm sncRNA populations over time in healthy adult men, each month for 6 months sperm samples were collected and perceived stress was assessed (Morgan et al., 2020). Five miRNA and four tRNA in sperm fluctuated with perceived stress in a dynamic manner, suggesting that they were responsive to stress. This means that these sncRNA might be able to signal stress in the environment, potentially at conception. Two of the sperm sncRNA identified as stress-responsive have been associated with childhood trauma in a recent study examining plasma sncRNA (Van der Auwera et al., 2019). Identifying sncRNA that respond to stress in human sperm is a first step to translating research from animals to humans. While no human studies have prospectively examined whether stress-induced sperm epigenetic changes are associated with offspring outcomes, research in animals suggests that they may be.

### Animal Models

Studying paternal transmission of preconception stress experiences in animal models eliminates major complications of studying this in either female animals (e.g., the fetoplacental environment) or humans (e.g., parental behavior). Male rodents, especially inbred mouse strains, are not typically involved in offspring rearing, allowing researchers to isolate the effect of paternal preconception stress on offspring outcomes. Studies in male animals provide the clearest evidence of germ cell epigenetic transmission of stress experience.

One model of early life stress uses maternal separation combined with unpredictable maternal stress (MSUS) from postnatal days 1 to 14. Four studies using this paradigm found that paternal early life stress exposure was associated with depressive-like behavior in F1 female offspring as well as behavioral despair and reduced fear in F1 offspring of both sexes (Franklin et al., 2010; Gapp et al., 2014, 2018; Bohacek et al., 2015). All four studies identified changes in sperm DNA methylation or sncRNA content, including methylation of genes that regulate the stress response and impart vulnerability to depression-like behavior, at least in mice (Franklin et al., 2010; Valverde and Torrens, 2012; Gapp et al., 2014, 2018; Bohacek et al., 2015). Paternal preconception exposure to MSUS was associated with physical health-related outcomes in offspring (Gapp et al., 2014, 2018). Although early life stress-exposed male mice had normal glucose regulation, their offspring had lower basal and stress-stimulated glucose levels (Gapp et al., 2014). Their offspring also showed signs of hypermetabolism as their body weight was lower despite higher caloric intake. Paternal preconception exposure to MSUS was also associated with worse neural and cognitive outcomes in sires and offspring (Bohacek et al., 2015). Both showed impaired synaptic plasticity and long-term memory. Sperm of F0 males and the hippocampus of F1 offspring showed reduced DNA methylation at the *Prkcc* promotor, a gene implicated in synaptic plasticity and memory performance.

In a separate model, adolescent male mice exposed to chronic social instability stress for 7 weeks showed reductions in the same sperm miRNA (miR-449a and miR-34c) as men exposed to ACEs in the study described previously (Dickson et al., 2018). Importantly, miR-449a and miR-34c are among a small set of miRNA that are not present in oocytes but are delivered to them via sperm during fertilization, suggesting that these miRNA may influence subsequent generations by altering early development (Yuan et al., 2016).

One study tested whether chronic variable stress experienced for 6 weeks, either during puberty or in adulthood, differentially affected F0 sperm miRNA content and F1 offspring stress reactivity (Rodgers et al., 2013). Regardless of when F0 males were exposed to stress, F0 sperm exhibited elevated levels of miRNA, nine that were robustly increased, and F1 offspring showed a significantly blunted HPA stress response to an acute stressor as well as gene set enrichment in stress regulatory brain regions, including the paraventricular nucleus and bed nucleus of stria terminalis (Rodgers et al., 2013; Chan et al., 2020).

In another stress model, adult male mice subjected to daily restraint stress for 2 weeks were bred and then physical health-related outcomes were measured in the offspring (Wu et al., 2016). F0 stress-exposed males exhibited a reduced body weight gain, elevated blood glucose levels, and a two-fold increase in sperm DNA methylation of the *Sfmbt2* promotor compared to control males (Wu et al., 2016). F1 offspring showed a similar increase in blood glucose, an effect that was more pronounced in male than female offspring. The increase in blood glucose may be explained by the fact that follow-up experiments in males showed that they had elevated expression of phosphoenolpyruvate carboxykinase, an enzyme involved in gluconeogenesis, a process by which glucose is generated from fats and proteins instead of carbohydrates.

### Factors Critical to Producing Molecular Changes in Sperm

#### Stress Hormones

One key assumption in this literature is that heightened exposure to glucocorticoids, both in terms of overall levels and length of exposure, during stress drives lasting epigenetic changes associated with germ cells. Several studies have isolated the specific role of glucocorticoids in this process by either administering synthetic glucocorticoids or utilizing corticosterone treatment. Overall, these studies found that synthetic glucocorticoids and corticosterone treatment were associated with epigenetic alterations in sperm and phenotypic changes in the offspring that mirrored those of paternal stress models (Petropoulos et al., 2014; Short et al., 2016; Wu et al., 2016; Chan et al., 2020). One study further showed that glucocorticoid antagonists given to males during a 14-day period of stress exposure blocked hypermethylation patterns in sperm as well as transmission of the stress-associated phenotypic changes in their offspring, supporting a role of the glucocorticoid receptor in this programming (Wu et al., 2016).

#### Stress Recovery Period

Exposure to stress or stress hormones are similar in that they do not immediately impart epigenetic changes on sperm. Two studies showed that changes in sperm miRNA content and DNA methylation marks are only detected after a period of recovery from stress or after ceasing synthetic glucocorticoids (Petropoulos et al., 2014; Chan et al., 2020). For example, in one study, sperm miRNA expression showed only minor differences 1 week after chronic stress ended but dramatic differences 12 weeks after stress cessation, and this coincided with the timeline required for transmission of the blunted HPA stress axis phenotype to the F1 offspring (Chan et al., 2020). Overall, a stress recovery period seems to be necessary to alter sperm epigenetic markers and the offspring phenotype. The stress recovery period may serve as a cellular establishment of a new allostatic set point necessary for transcriptional responses, changes in secreted signals, and altered epigenetic marks in germ cells.

### Evidence That Molecular Changes in Sperm Matter

Epigenetic alterations associated with sperm are assumed to be causal in the changes detected in the F1 offspring phenotype, but only some studies have actually isolated the role of stress-induced changes in sperm in producing offspring phenotypic changes. *In vitro* fertilization and intracytoplasmic sperm injection methods have been used to eliminate potential confounds – namely, the possibility that mating with a stressed male affects the stress physiology and maternal behavior of the female, which could be driving the observed effects (Dias and Ressler, 2014; Wu et al., 2016; Chan et al., 2020). Strong causal evidence comes from studies showing that a similar pattern of offspring phenotypic changes emerge regardless of whether offspring are derived from natural breeding, *in vitro* fertilization, or intracytoplasmic sperm injection (Dias and Ressler, 2014; Wu et al., 2016; Chan et al., 2020; but see also Dietz et al., 2011). In order to implicate sperm miRNA content specifically in driving offspring outcomes, researchers have directly microinjected into zygotes miRNA from stress-exposed sperm or mimics of identified miRNA changed in stress-exposed sperm (Gapp et al., 2014, 2018; Rodgers et al., 2015). These experiments substantiate the claim that stress-altered sperm miRNA are responsible for transmitting offspring phenotypic changes.

### How Stress May Alter sncRNA Content in Sperm

**FIGURE 2:**
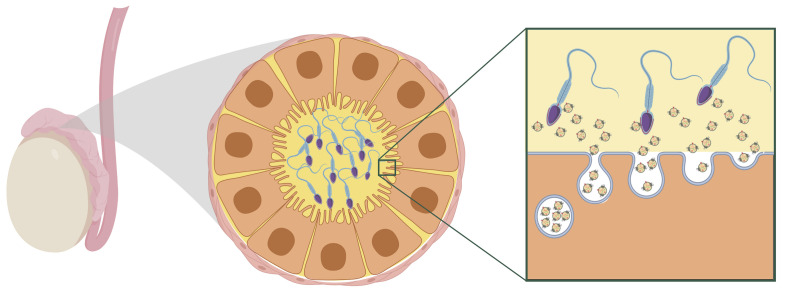
Hypothesized model of maturing sperm interacting with extracellular vesicles within the caput epididymal lumen. Reprinted from Chan et al. (2020). Reproductive tract extracellular vesicles are sufficient to transmit intergenerational stress and program neurodevelopment. Nature Communications. 11:1499. https://creativecommons.org/licenses/by/4.0/.

An important missing piece of the puzzle has been understanding how paternal stress leads to changes in sperm sncRNA content when sperm are transcriptionally inert. Recently, however, evidence has emerged that illuminates a potential mechanism. Epididymal epithelial cell extracellular vesicles in the caput epididymis encode environmental challenges through changes in their sncRNA content. As sperm transit through the epididymis during maturation, they gain sncRNA from extracellular vesicles (Belleannée et al., 2013; Nixon et al., 2015; Sharma et al., 2016). The current understanding is that the somatic epididymal epithelial cells maintain epigenetic changes following stress and that this affects the sncRNA content of secreted extracellular vesicles. Thus, these cells are able to encode and signal stress to sperm even after the stress exposure subsides (Morgan et al., 2019; see Figure 2).

A recent study in mice provides some of the first evidence that extracellular vesicles secreted from epididymal epithelial cells are able to transmit stress signals to sperm and reproduce a specific offspring phenotype of paternal stress (Chan et al., 2020). Epididymal epithelial cells in culture were chronically treated with either corticosterone or vehicle for three days, and extracellular vesicles recovered from the media of these cells were collected 8 days later, following three media changes occurring after treatment. Sperm collected from stress-naïve male mice were incubated with extracellular vesicles from either the corticosterone or vehicle treatment. Sperm were then injected into control oocytes via intracytoplasmic sperm injection and transferred into the right or left uterine horn of recipient females to control for the intrauterine environment. In embryos derived from sperm exposed to corticosterone-treated extracellular vesicles, the developing brain at mid-gestation showed significant changes in genes underlying synaptic signaling and neurotransmitter transport. As adults, offspring generated from the corticosterone-treated extracellular vesicles phenocopied the blunted corticosterone response of offspring produced from naturally bred stress-exposed males. Importantly, this study provides evidence for somatic to germ cell transmission of a stress signal. In this model, exposure to stress is signaled in an existing signaling pathway along the reproductive tract. This mechanism promotes lasting programmatic change in a way that allows the paternal environmental experience to be transmitted between generations but does not itself require perpetuation of an epigenetic mark following conception.

## Discussion

**FIGURE 3:**
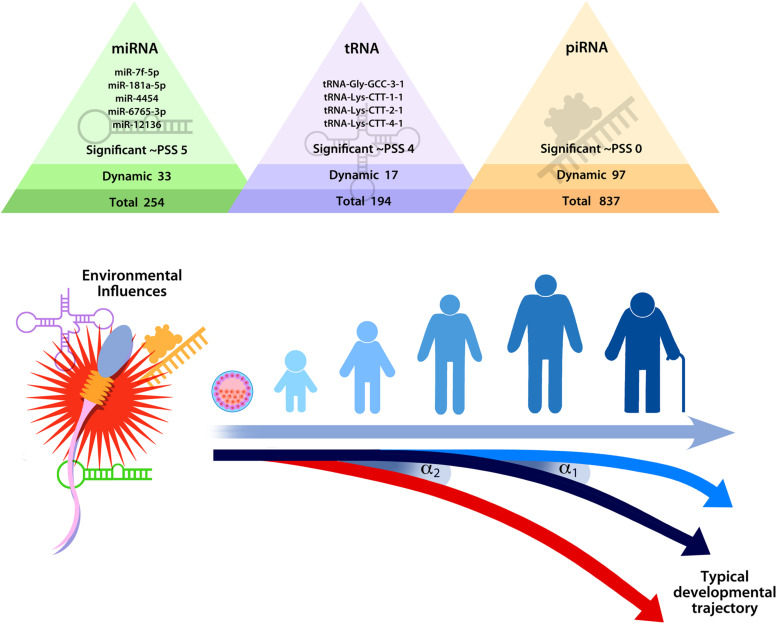
Hypothesized model of the impact of stress on sperm small non-coding RNA content on developmental trajectories, highlighting the fact that relatively minor changes in sperm small non-coding RNA (as indicated by α1 or α2) could shift the timing of developmental events (e.g., embryo division and implantation) in ways that produce significant differences over time. Reprinted from Morgan et al. (2020). Repeated sampling facilitates within- and between-subject modeling of the human sperm transcriptome to identify dynamic and stress-responsive sncRNAs. Scientific Reports. 10:17498. https://creativecommons.org/licenses/by/4.0/.

Evidence supporting epigenetic transmission of stress is still emerging, and many important questions remain. Although studies have found that paternal preconception stress is associated with epigenetic alterations in sperm, the value of these differences remains largely unknown. Even as some evidence has confirmed that epigenetic modifications identified in sperm alter offspring development in a meaningful way, the biological mechanism remains unclear for how these epigenetic changes impact offspring development. The prevailing thought is that sncRNA alter the developmental trajectory of the offspring by initiating a cascade of molecular events that propagate the stress signal, shifting the timing of developmental events and guiding development toward a set of neurobiological, physiological, and behavioral traits that are more adaptive in a stressful environment (Morgan et al., 2019, 2020; see Figure 3).

Sperm sncRNA play a critical role in early zygote and embryo development (e.g., Liu et al., 2012; Sendler et al., 2013; Yuan et al., 2016; Guo et al., 2017; Conine et al., 2018; Godia et al., 2018). Even relatively minor modifications in sperm sncRNA content could shift timing of developmental events in a meaningful way that could produce significant phenotypic changes in the offspring. A feasible example of how sncRNA might affect development comes from consideration of protogenin, a gene that regulates early embryonic transitions (Wong et al., 2010; Chen and Wang, 2020). Two of the stress-responsive sncRNA identified in human sperm target protogenin (Morgan et al., 2020). Therefore, it is conceivable that changes in the levels of sperm let-7f-5p and miR-181a-5p at conception could dynamically regulate protogenin expression and impact the rate of zygotic and embryonic division. If stress-induced epigenetic modifications delivered by sperm indeed affect the rate of zygotic and embryonic development, then this would fit within the framework established by previous research. Specifically, early life stress effects on developmental programming that prepare the offspring for adverse conditions may originate preconception.

Though the cascade of developmental changes that result from the epigenetic signal may be intended to be adaptive, these phenotypic changes may nevertheless have implications for risk and resilience to disease in the offspring. However, it is important to emphasize that epigenetic changes are not deterministic, and the majority of the studies to date only show “differences,” not a disease. Epigenetic effects on the offspring may tip the scale toward risk or resilience, but the emergence of disease relies on the interplay between genetic and environmental risk factors.

More studies are needed before firm conclusions can be drawn. For research in females, strong methodologies are needed to contend with critical confounds – namely the intrauterine environment and maternal behavior. For example, in humans, one study showed that the intrauterine environment can transmit maternal preconception stress to offspring via greater placental corticotropin-releasing factor during pregnancy (Moog et al., 2016). In animal studies, transferring the embryo to a surrogate female is a powerful method that allows researchers to disentangling preconception effects on oocytes from effects *in utero* (Tsai et al., 2020). Likewise, preconception stress in rats affects the quality of maternal behavior, another potential confounding factor (Keller et al., 2019). Cross-fostering newborns with a surrogate mother allows biologically driven effects of maternal preconception stress to be separated from socially driven effects.

Broadly, future research must consider important moderators in order to draw conclusions that are more nuanced rather than oversimplified. Almost no research has examined whether the type and timing of the stressor is critical to alterations in the offspring phenotype. In the current literature, the impact of stressor type and timing is difficult to disentangle because studies use different stress paradigms, assess different outcomes, and do not always specify the developmental time period of stressor exposure. To our knowledge, only one study has examined the impact of developmental timing within the same experiment (Rodgers et al., 2013). Although this study did not observe phenotypic differences in the offspring of male mice depending on whether they were exposed to stress in adolescence or adulthood, one study in humans suggests that developmental timing may matter (Bygren et al., 2001). In this retrospective study, nutritional challenges during the grandfather’s preadolescent slow growth phase were associated with changes in the grandson’s longevity. This finding suggests that future studies should consider the impact of stressor type and timing. In addition, many studies report sex differences, suggesting that parent and infant sex may be important moderators of epigenetic germline transmission of stress and that future research should test for sex differences (Shachar-Dadon et al., 2009; Franklin et al., 2010; Saavedra-Rodríguez and Feig, 2013; Bohacek et al., 2015; Zaidan and Gaisler-Salomon, 2015; Bock et al., 2016; Wu et al., 2016; Cissé et al., 2020). Finally, prospective longitudinal studies are needed in order to translate hypotheses generated in animal models to humans. Only then will we be able to differentiate the effects of different types of real-life stress exposures on germ cell epigenetic changes in humans.

## Author Contributions

KD drafted the manuscript under the guidance of TB and CNE. TB and CNE provided critical revisions and feedback. All authors contributed to the article and approved the submitted version.

## Conflict of Interest

CNE consults for Sage Therapeutics and Asarina Pharma and is an investigator for a multisite clinical trial conducted by Sage Therapeutics. The remaining authors declare that the research was conducted in the absence of any commercial or financial relationships that could be construed as a potential conflict of interest.
